# Mechanical and *In Vitro* Biological Performance of Graphene Nanoplatelets Reinforced Calcium Silicate Composite

**DOI:** 10.1371/journal.pone.0106802

**Published:** 2014-09-17

**Authors:** Mehdi Mehrali, Ehsan Moghaddam, Seyed Farid Seyed Shirazi, Saeid Baradaran, Mohammad Mehrali, Sara Tahan Latibari, Hendrik Simon Cornelis Metselaar, Nahrizul Adib Kadri, Keivan Zandi, Noor Azuan Abu Osman

**Affiliations:** 1 Department of Mechanical Engineering and Center of advanced Material, University of Malaya, Kuala Lumpur, Malaysia; 2 Tropical Infectious Diseases Research and Education Centre (TIDREC), Department of Medical Microbiology, Faculty of Medicine, University of Malay, Kuala Lumpur, Malaysia; 3 Department of Biomedical Engineering, Faculty of Engineering, University of Malaya, Kuala Lumpur, Malaysia; Universidade Federal do ABC, Brazil

## Abstract

Calcium silicate (CaSiO_3_, CS) ceramic composites reinforced with graphene nanoplatelets (GNP) were prepared using hot isostatic pressing (HIP) at 1150°C. Quantitative microstructural analysis suggests that GNP play a role in grain size and is responsible for the improved densification. Raman spectroscopy and scanning electron microscopy showed that GNP survived the harsh processing conditions of the selected HIP processing parameters. The uniform distribution of 1 wt.% GNP in the CS matrix, high densification and fine CS grain size help to improve the fracture toughness by ∼130%, hardness by ∼30% and brittleness index by ∼40% as compared to the CS matrix without GNP. The toughening mechanisms, such as crack bridging, pull-out, branching and deflection induced by GNP are observed and discussed. The GNP/CS composites exhibit good apatite-forming ability in the simulated body fluid (SBF). Our results indicate that the addition of GNP decreased pH value in SBF. Effect of addition of GNP on early adhesion and proliferation of human osteoblast cells (hFOB) was measured in vitro. The GNP/CS composites showed good biocompatibility and promoted cell viability and cell proliferation. The results indicated that the cell viability and proliferation are affected by time and concentration of GNP in the CS matrix.

## Introduction

Calcium silicate, (CaSiO_3_, CS), has been investigated as a bioactive biomaterial for tissue repair and replacement due to its osseointegration properties [1]–[5]. However, the extensive use of calcium silicate is still limited by its brittle nature, low fracture toughness and poor wear resistance. Thus, toughening of CS with a second phase such as yttria stabilized zirconia, Alumina, Ti_3_SiC_2_ and titanium has been explored to overcome the deficiencies of pure CS [6]–[9]. On the other hand, CS is difficult to densify by an ordinary sintering technique, so that the relative densities of the sintered β-calcium silicate (low-temperature phase) and α-calcium silicate (high-temperature phase) reported to date are below 90%, which further compromises the mechanical properties. Few of the materials that have been attempted for CS-based bioceramics have sufficient mechanical properties and favourable biocompatibility at the same time.

Rafiee *et al.* [10], [11] have shown that graphene reinforcement in ceramic-matrix composites can provide an excellent toughness, inhibiting the crack propagation and improving mechanical properties. Graphene has a very high electron mobility at room temperature (250,000 cm^2^/Vs), exceptional in-plane thermal conductivity (5000 Wm^−1^ K^−1^) and superior mechanical properties with a Young's modulus of 1 TPa and high tensile strength (130 GPa) [12], [13]. Its potential applications include single molecule gas detection, transparent conducting electrodes, nanofluids and energy storage devices such as supercapacitors, lithium ion batteries and phase change materials [12], [14]–[19]. Compared to single layer graphene, graphene nanoplatelets (GNP) are less prone to agglomeration and entanglement due to increased thickness of GNP. Because of these properties, GNP has been used as reinforcement in composite materials. Recently, some studies were conducted on Alumina [20], silicon nitride [11], and tantalum carbide [21] matrices reinforced with GNP in order to improve mechanical properties of the ceramics. More recently, Zhao *et al.* [22] used hot pressing (HP) to prepare graphene nanoplatelet (GNP)/biphasic calcium phosphate (BCP) composite, and a 76% increase in fracture toughness was obtained. They employed aqueous colloidal processing methods to obtain a uniform and homogenous dispersion of GNP and BCP ceramic particles. Zhang *et al.* [23] have reported that the fracture toughness of a GNP-reinforced hydroxyapatite (HA) matrix can be improved by up to 80% by spark plasma sintering (SPS) and they have also shown that the added GNP contributes to the improvement of both osteoblast cell adhesion and apatite mineralization. Moreover, recent studies have shown that graphene and graphene-based composites possess a series of merits, e.g., are non-toxic for human osteoblasts and mesenchymal stromal cells [24], suitable for adhesion and proliferation of osteoblasts [25], have excellent antibacterial property [26], and the ability of apatite mineralization [27]. To the best of our knowledge, no study has yet explored the mechanical and biological properties of a free-standing graphene-calcium silicate composite.

Therefore, the aim of this study is to investigate the potential of using GNP as reinforcement to CS for load-bearing orthopaedic applications. CS and GNPs/CS composites are produced using hot isostatic pressing (HIP), and the mechanical properties of the sintered samples and apatite formation in a simulated body fluid (SBF) are evaluated. In addition, detailed *in vitro* experiments, such as cell adhesion and cell proliferation (MTT) are analysed in order to explore the capabilities of such materials to be successfully applicable as biomaterials in tissue engineering applications.

## Materials and Methods

### Material preparation

Calcium silicate (CaSiO_3_) powders were synthesized through a chemical precipitation method using reagent-grade calcium nitrate (Ca (NO_3_)_2_.4H_2_O) and reagent-grade sodium silicate (Na_2_SiO_3_·9H_2_O) as precursors (Sigma-Aldrich, Inc., St. Louis, MO, USA). The synthesis process was similar to the procedure described by Long *et al.* [28] and is not shown here for brevity. Precipitates were washed thoroughly with distilled water, dried at 90°C for 24 h, and then calcined at 800°C for 2 h. Finally, the powders were ball milled for 12 h. The obtained powder was β- CaSiO_3_ with an average particle size of about 0.5 µm. Graphene nanoplatelets (GNP) (XG Sciences, USA) have an average thickness of a 2 nanometers and a particle diameter of less than 2 microns with a specific surface area of 500 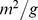 (Grade C) were first dispersed in cetyltrimethylammonium bromide (CTAB) and sonicated for one hour. CS powder was then added and the mixture was sonicated for another 30 min. The suspension was then mixed using zirconium oxide balls (Retsch GmbH, Haan, Germany) in a horizontal ball mill (9VS, Pascall Engineering Co. Ltd, Suffolk, UK) for 12 h. The milled slurry mixture was dried at 100°C in an oven for 2 days. The chosen composites for the purpose of this study were pure CS and GNP/CS composites with GNP contents of 0.5, 1.0, 1.5 and 2.0 wt%.

### Hot isostatic pressing (HIP) processing

Green bodies (5 and 10 mm diameter) of cylindrical shape were formed by uniaxial pressing at 250 MPa. The sintering procedure was completed at 1150°C and 160 MPa pressure applied for 1 h in high-purity argon gas by hot isostatic pressing (American Isostatic Presses, Inc., USA). The heating and cooling rates did not exceed 5°C/min. It is noteworthy, in order to eliminate the CTAB, the compacted samples were heated to 500°C for 1 h during HIP processing. The sintered samples were polished with SiC abrasive papers (up to 1200 grit size), and polished to mirror finish using diamond powder of various grades from 15 to 0.25 µm in an auto polisher (laboforce-3, Struers). The samples were also thermallyetched at 1050°C for 30 min in a muffle furnace for grain size determination.

### Structural characterization

The microstructure of the powder mixture (GNP, CS and GNP/CS) was observed using a high-resolution FEI Quanta 200 F field emission scanning electron microscopy (FESEM). Observation of the GNP/CS composites interface in near atomic-scale was carried out under a transmission electron microscopy (TEM, Zeiss Libra 120). For the TEM characterization, the sample preparation consisted of dispersing the powder in ethanol, placing onto the micro grid, and letting the solvent evaporate. The X-Ray diffraction (XRD) pattern of the powders and composites were obtained using an automated X-ray powder diffractometer (XRD, PANalytical's Empyrean) with a monochromated CuKα radiation (λ = 1.54056 Å), which was operated at 45 kV and 40 mA with a step size of 0.026 deg and a scanning rate of 0.1 deg s^−1^ in the 2θ range of 20 to 60 deg. Energy dispersive X-ray analysis (EDS) using on EDX-System (Hitachi, S-4800) instrument attached to the FESEM to investigate the elemental composition of the samples. Raman spectra were obtained using a Renishaw Invia Raman Microscope using laser excitation at 514 nm. The Brunauer-Emmett-Teller specific surface areas of the samples were evaluated on the basis of nitrogen adsorption isotherms measured at 77 K using a BELSORP-max nitrogen adsorption apparatus (Japan Inc.). The densities of sintered samples were measured by the Archimedes method. The rule of mixtures was used to calculate the theoretical densities of the composites, based on weight percentage, using density values of 2.90 and 2.1 g/cm^−3^ for CS and GNP, respectively.

### Mechanical properties evaluation

Microhardness was measured using a Mitutoyo hardness tester (model AVK-C2, Mitutoyo, Kawasaki, Japan). A 1 kg Vickers load was applied to the polished samples with a loading time of 10 s. A total of 10 points were collected for each specimen. Nanoindentation experiments were conducted using a nanomechanical test system (Micro Materials Ltd. Wrexham, U.K.) employing Berkovich diamond tip with a radius of 20 nm using 100 mN in a load-controlled mode with a dwell time of 10 s. The indentation velocity was 3 nms^−1^. At least ten indentations were made to obtain an average value for each sample. Elastic modulus was calculated from the load–displacement unloading curves using the Oliver–Pharr method [29]. The fracture toughness was then calculated from the equation suggested by Anstis [30]:
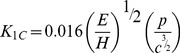
(1)


Where K_IC_ is the indentation toughness (MPa m^1/2^), 0.016 is the material-independent constant for a Vickers radial crack, *E* is the elastic modulus (GPa) from the nanoindentation experiments, *H* is the Vickers hardness (GPa), *P* is the indentation load (N), and *C* (m) is the half-length of the radial cracks on the surface after indentation.

Quantitative evaluation of the machinability of the GNPs/CS composites was performed by calculating the brittleness index (BI). The brittleness index has been determined by the following equation [31]

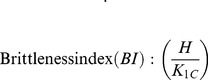
(2)


In the current study, the average values of H and K*_IC_* were used for calculation.

### Soaking in simulated body fluid

The bioactivity of the fabricated GNP/CS composites was evaluated by examining the formation of bone-like apatite on the samples in simulated body fluid (SBF), which was prepared according to Kokubo's method [32]. The as-sintered samples with a size of 3 mm thickness and 10 mm diameter were soaked in SBF at 37°C in a humidified atmosphere of 5% CO_2_ for 1, 3, 7 and 14 days, respectively, at a surface area to volume ratio of 0.1 cm^2^/mL. After soaking for various periods, the samples were removed from the SBF, gently rinsed twice with deionized water to remove SBF followed by drying in vacuum at 80°C. The soaked samples were characterized by XRD, and the surface was observed by FESEM.

### Cell attachment and proliferation assay

The human osteoblast cell lines (hFOB 1.19 SV40 transfected osteoblasts, ATCC, Rockville, MD, USA) were seeded on the sterilized pure CS and GNP/CS composites samples at a density of 1×10^4^ cells ml^−1^ in 96-well culture plates. Cells were maintained and propagated in DME/F-12 (HyClone, UT) cell culture medium supplemented with 10% fetal bovine serum (Gibco, NY), 100 U/mL penicillin and 100 µg/mL streptomycin at 37°C in a humidified atmosphere with 5% CO_2_ and cultured for 1,3 and 5 days. Proliferation of the cells cultured on the sterilized pellets (5 mm in diameter) was analysed using the methyl thiazole tetrazodium (MTT) assay. An MTT stock solution of 5 mg ml^−1^ (Sigma, St. Louis, MO, USA) was prepared by dissolving MTT in PBS, filtered through a 0.2 µm filter and stored at 4°C. Then the 96-well plate was removed from the incubator and 20 µl MTT stock solutions were added to each well. Cells were incubated for 4 h at 37°C in an atmosphere of 100% humidity and 5% CO_2_. After the incubation, the MTT solution was removed and replaced with 100 µl DMSO. At each culture period (1, 3 and 5 days), the samples were taken out and removed to new 24-well tissue culture plates. After being washed three times with PBS solution, cells were detached with trypsin/EDTA and stained with trypan blue, after which the living cells were counted with a hemocytometer (Becton Dickinson, Germany). Five samples of each composite were tested, and each test was carried out in triplicate.

### Cell morphology

For the purpose of FESEM and confocal laser scanning microscopy observation of the cells adhering on the surfaces of the samples, following incubation for 1,3 and 5 days, the cells were fixated at the surface of the specimens with 4% glutaraldehyde for 2 h at room temperature followed by washing the samples in PBS (0.1 M) for three times and dehydration with a series of graded ethanol/water solutions (40%, 50%, 60%, 70%, 80%, 90% and 3×100%, respectively). Then 0.5 ml hexamethyldisilazane (HMDS) was added to each well to preserve the original morphology of the cells, andthe test plates were kept in a fume hood to dry at room temperature.

### Confocal laser scanning microscopy

The specimens were first washed with 1X PBS before staining with 100 µg/ml of acridine orange (Sigma Aldrich, St. Louis, MO, USA) for 5 minutes at room temperature. Excess stain was removed by washing twice with 1X PBS for 10 minutes each. The stained cells were then analyzed using confocal microscopy (Leica TCS-SP5 II, Leica Microsystem, Mannheim, Germany) and the image processed with Leica LAS AF software.

### Statistical analysis

All data were expressed as the mean ± standard deviation (SD) and were analyzed using a one-way analysis of variance (ANOVA) and a Tukey–Kramer post hoc test. P<0.05 was considered statistically significant.

## Results and Discussion

### Powder processing

**Figure 1:**
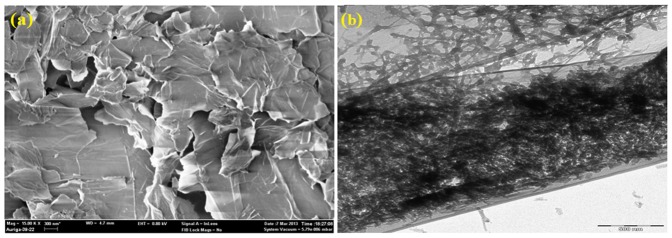
Micrograph of powders. (a) FESEM micrograph of wrinkled top surface of GNPs, (b) TEM of GNPs/CS powder mixture showing the CS particles are well-dispersed throughout the surface area of the graphene sheets.

The pristine GNP and 1 wt.% GNP/CS composite powders were analysed prior to sintering in order to evaluate the effectiveness of the mixing and processing. As revealed by TEM analysis, GNP exhibits a flake structure with various in-plane sizes and indicated well-ordered graphene layers (Fig. 1a). Moreover, the thickness of GNP is less than several nanometres. Fig. 1c shows the dispersion of GNPs, which were distributed homogeneously in the CS powder. In this experiment, we employed 1.0 wt% CTAB to CS powders and 1.0 wt% to GNP as the dispersant to disperse GNP in the CS powder. Walker *et al*. [11] reported that the dispersion of GNP using CTAB occurs because the hydrophobic GNP is attracted to the hydrophobic tails of the surfactant. As a result, GNP is covered in positively charged surfactant molecules. On the other hand, CS generally has a negative charge due to a deficiency of calcium ions, resulting in CS powders that are attracted to the GNP surface owing to electrostatic interaction once GNP suspension was mixed, hence avoiding the agglomeration of the graphene nanoplatelets and leading to uniform dispersion of GNP in the CS powders.

### Microstructural characteristics

**Figure 2:**
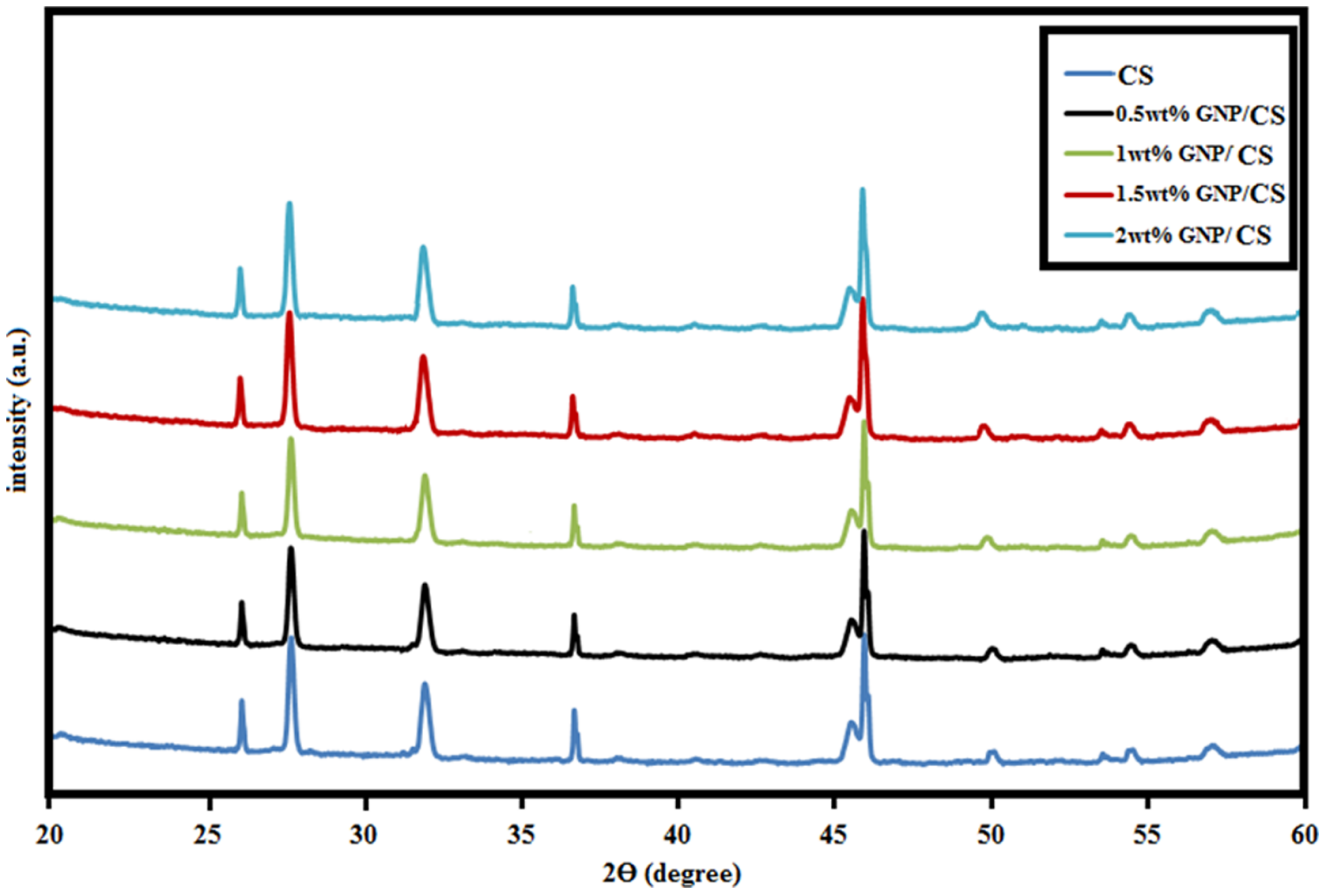
XRD results of pure CS and GNP/CS composites sintered at 1150°C by HIP.

The XRD patterns of the GNPs/CS composites sintered at 1150°C by HIP are shown in Fig. 2. Only α-CaSiO_3_ phase existed (Standard cards no JCPD 31-0300) in pure CS and GNPs/CS. The patterns are similar, indicating that the incorporation of GNP has no effect on the crystal phase composition of CS. Meanwhile, GNPs are present in minor quantities and are difficult to detect by XRD. The pristine GNPs and 1 wt%. GNPs/CS before and after HIP consolidation were analysed using Raman spectroscopy to verify the existence, and evaluate the structure of GNP.

**Figure 3:**
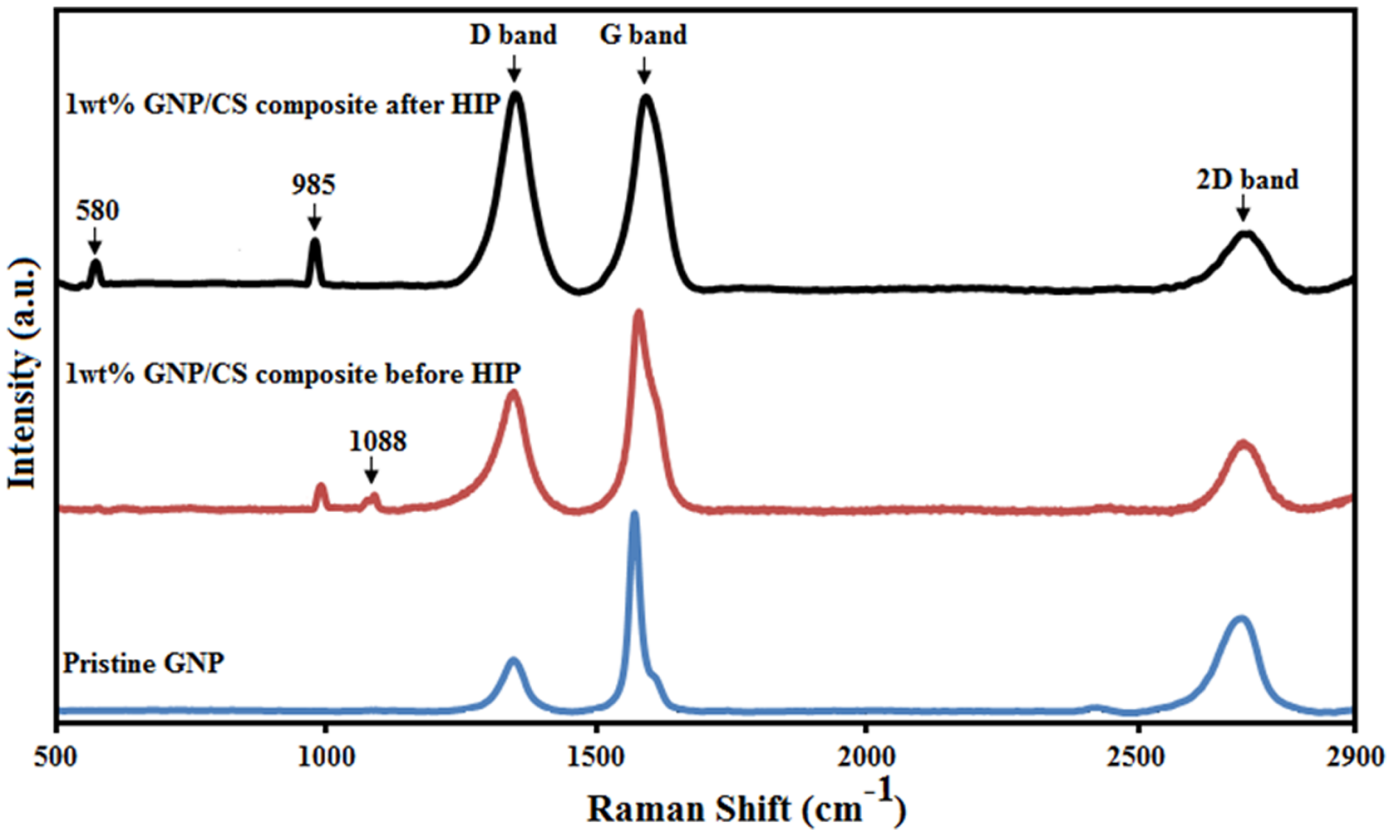
Raman spectra of pristine GNP, GNP/CS composite (1 wt.% GNP) before HIP and GNP/CS composite (1 wt.% GNP) after HIP.

Fig. 3 and Table 1 display the presence of D, G and 2D peaks. The D peak was associated with the presence of disorder in the aromatic structure or the edge effect of graphene, the G peak is from in-plane C-C bond stretching in graphene, and the 2D peak is related to the thickness and also used to determine the number of graphene layers [23], [33]. The presence of G and 2D peaks in the GNP/CS composite confirms the retention of GNP even after HIP consolidation. The pristine GNP showed a D-band around 1347 cm^−1^, G-band around 1570 cm^−1^ and 2D peak ∼2690 cm^−1^. After mixing of GNP and CS powders by ball milling process, D and 2D peaks have shifted to higher wave numbers of 1580 cm^−1^ and 2700 cm^−1^, respectively. Moreover, It can be seen that the D, G, 2D peaks in the 1 wt.% GNP/CS composite after sintering have shifted to higher energies, especially the G band exhibited a blue-shift from 1570 to 1595 cm^−1^ after HIP. The spectral blue-shifts could be ascribed to the disturbing of the graphene structure caused by the compressive stresses acting on GNP, incurred during thermal contraction of CS matrix [34]. The intensity ratio of the D to G-bands (*I*_D_/*I*_G_) is a measure of the degree of disorder, the larger the ratio the more defects present [35]. As shown in Fig. 3 and Table 1, the *I*_D_/*I*_G_ ratio of pristine GNP, 1 wt.% GNP/CS powder and 1 wt.% GNP/CS composite after HIP were 0.26, 0.59 and ∼1, respectively, implying that the ball milling and HIP process introduces structural defects into GNPs. The ball milling of GNP/CS powders leads to strong interactions between GNP and CS particles. These interactions appear to have adverse effects on the GNP resulting in higher *I*_D_/*I*_G_ ratio indicating partial loss of graphene-like structure. Nevertheless, the presence of G and 2D peaks in the GNP/CS powder exhibited the existence of graphene-like structure. Moreover, the *I*_2D_/*I*_G_ intensity ratio of 1 wt.% GNP/CS powder before HIP processing decreased from 0.48 to 0.34 compared to pristine GNP, indicating an increase in the number of graphene layers due to mixing process [36]. Furthermore, our results indicate that the *I*_2D_/*I*_G_ values of 1 wt.% GNP/CS powders and GNP/CS slightly decreased from 0.34 to 0.3, illustrating an increasing number of graphene layers after HIP process [37]. Thus, Raman spectroscopy demonstrates that the GNP structure is retained after HIP consolidation. The spectrum of the GNP/CS powder before sintering exhibits peak representing the β-CS phase at 1088 cm^−1^, which can be attributed to the Si−O−Si asymmetric stretching mode (ν_as_(Si−O−Si)) and 985 cm^−1^ is attributed to Si-O stretching vibration [38]. In addition, two characteristic Raman peaks for the α-CS at 580 and 985 cm^−1^ were detected in 1 wt.% GNP/CS composites after the HIP process and are attributed to Si-O-Si bending vibration and at the Si-O stretching vibration, respectively [39], [40].

**Table 1:** Peak position of the D and G bands and intensity ratios of *I*_D_/*I*_G_ and *I*_2D_/*I*_G_.

Samples	D band (Rman shift)	G band (Rman shift)	2D band (Raman shift)	*I*_D_/*I*_G_	*I*_2D_/*I_G_*
GNP	1347	1570	2690	0.26	0.48
1 wt% GNP/CS before HIP	1347	1580	2700	0.59	0.34
1 wt% GNP/CS after HIP	1350	1595	2700	1	0.3

### Physical and mechanical properties

Pure CS and GNP/CS achieved high degrees of densification after HIP, with relative densities ranging from ∼90% to 98.5%.

**Figure 4:**
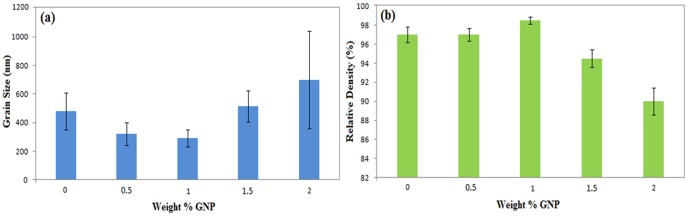
The effect of the GNP on the grain size and densification. (a) Grain Size vs. GNP content. (b) Densification (relative to theoretical density) vs. GNP content.

**Figure 5:**
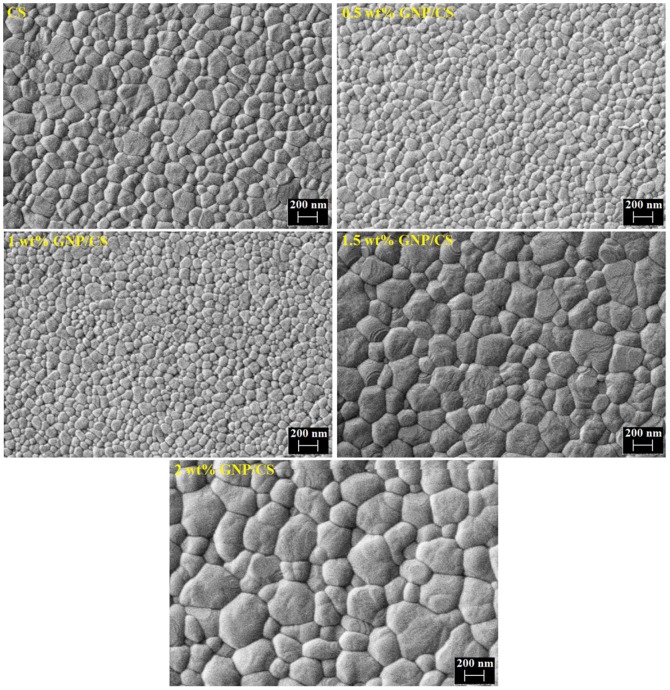
FESEM images of thermally etched surfaces for pure CS and GNP/CS composites.

Fig. 4b is a plot of the relative density of the GNP/CS composites, shown as a function of the GNP concentration. The addition of GNP influences the density of the composite; the pure CS sample reached a density of ∼97% whereas 1 wt% GNP containing CS has a density of ∼98.5%, however when the content GNP increases further (1.5 and 2 wt%) the relative density of the composite decreases. GNP has a much higher thermal conductivity (5300 W/mK) [41] than CS, which makes it possible for the composite to have a more uniform distribution of the temperature during sintering. The consistent heating of the powders leads to improved densification. However, our results indicate that the incorporation of 1 wt% GNP into CS achieved the highest density. Fig. 5 shows the polished and thermally etched surfaces of GNP/CS composites. In addition, the mean grain size with varying GNP content is plotted in Fig. 4a. As can be seen from the FESEM images and the grain size analysis, the different amounts of GNP have an effect on the grain size. Significant grain refinement occurred for 0.5 and 1 wt% GNP/CS composites, where the grain sizes were reduced by over 40% relative to the pure CS. A recent study by Nieto *et al.* [21] has shown that the GNP can influence and reduce the grain size of tantalum carbide.

This is attractive for ceramics, as grain size refinement could simultaneously increase fracture toughness and hardness of the ceramic due to the change of cracking mode from transgranular to intergranular and the deflection of propagating cracks [42]. The concentration of GNP in 1 wt% GNP/CS is satisfactory for grain refinement and to hinder grain growth throughout the structure. The generated fine-grained structure is due to the grain boundary pinning action of GNP. Moreover, the high thermal conductivity and high surface area of GNP allow for strong interfacial bonding between CS grains and GNP, whichminimizes porosity formation. On the other hand, in high amount of GNP (1.5 and 2 wt%), the grain sizes increased. Further increase in GNP content causes agglomeration, which may lead to increasing thermal conductivity but not necessarily of providing effective grain pinning and wrapping.

**Figure 6:**
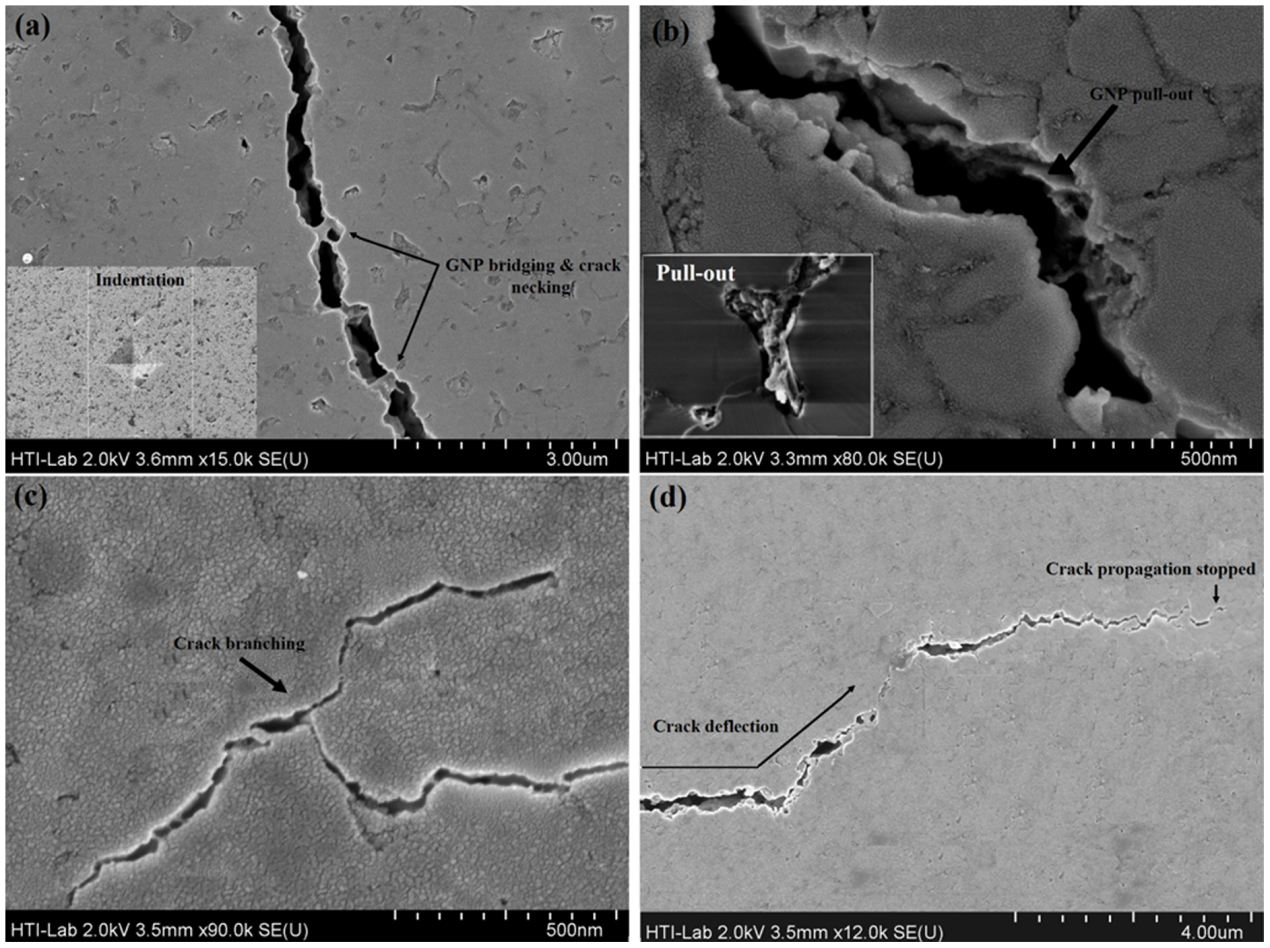
GNP toughening mechanisms in CS. (a) Micro hardness indent resulting in the creation of radial cracks (inset image). Closer examination of the radial cracks revealed GNP bridging, (b) sheet pull-out, (c) crack branching, (d) crack deflection.

The mechanical properties of pure CS and the GNP reinforced CS composites are compiled into Table 2. The modulus of elasticity of pure CS (110±9) in the high-temperature phase (α-CS) is at the higher end of values reported in our previous work and in the literature [6], [9], [28]. The modulus of elasticity increased slightly for 0.5 and 1 wt.% GNP/CS composites, but decreased for 1.5 and 2 wt.% GNP content. The increase in the modulus of elasticity of 0.5 and 1 wt.% GNP/CS samples is due to the high modulus of elasticity of graphene, the high relative density, and smaller grain sizes. On the other hand, porosity has been reported to be a major factor governing the modulus of elasticity for some ceramic materials, with greater porosity correlated with a lower modulus of elasticity [43]. This explains the reduction of the modulus of elasticity in 1.5 and 2 wt.% GNP/CS samples. The modulus of elasticity of human cortical bone is reported to be in the range of 15–25 GPa [44], while the modulus is much higher for consolidated pure CS and our composites. A mismatch of the modulus of elasticity at the bone–implant interface might pose a risk of fracture or delamination of the implant [44]. Nevertheless, the osseointegration ability of CS creates strong bonding at the CS–bone interface, thus decreasing the chance of fracture and delamination. Likewise, an increase in the modulus of elasticity directly influences the improvement in the fracture toughness in ceramic-based composite materials [42]. The hardness was assessed using Vickers indentation method at load 1 Kg, and illustrated in Table 2. It is clearly seen that hardness for 1 wt.% GNP/CS composite show improvements of ∼30%, as compared to pure CS. Our results indicate that addition of up to 1 wt.% GNP improves the hardness of CS because of strengthening of the matrix and grain size refinement, both of which prevented plastic deformation. It is noteworthy that the hardness is reduced at 1.5 and 2 wt.% GNP/CS composites due to increased porosity and growing average grain size (Fig. 5). In our study, the addition of GNP to CS results in improvement in the indentation fracture toughness as shown in Table 2. The fracture toughness is increased by ∼130% in the 1 wt.% GNP/CS composite. The increase in toughness correlates with increasing GNP content, but this trend does not continue for 1.5 and 2 wt.% GNP due to the high porosity, which is believed to provide nucleation sites for fracture and to weaken the strength of the ceramic composites. This may explain the fact that the addition of more than the optimum amount of GNP led to less strong composites [20]. The surfaces of the GNP/CS composites were analysed using FESEM in order to develop a comprehensive understanding of the contribution of the added GNP to the improved fracture toughness. As depicted in Fig. 6. GNP toughening mechanisms in CS, (a) Micro hardness indent resulting in the creation of radial cracks (inset image). Closer examination of the radial cracks revealed GNP bridging, (b) sheet pull-out, (c) crack branching, (d) crack deflection. Fig. 6a illustrates the indentation-induced crack propagation on the polished surface of GNP/CS composite, when a crack propagates and meets with GNP, which acts as a bridge and restricts the widening of the crack. GNP bridges need more energy for opening up of the cracks and this caused toughening. Hence, the crack propagating through CS gets restricted when it comes in the proximity of GNP, and consequently a higher energy is required for GNP debonding. Other studies have also observed that graphene bridging is an effective mechanism for the toughening of ceramics–graphene composite structure [11], [23], [45]. Fig. 6b shows that once a crack propagates through the CS matrix and finds a GNP across its path, the ridges on the GNP surface may be the first to experience pull-out resulting in energy dissipation, because of binding and friction which, subsequently, leads to toughening. Moreover, probing within the cracks (inset image), one can observe direct evidence of GNP pull-out and GNP sheets that are bridging the cracks. Fig. 6c and 6d show the intrinsic GNP branching and deflection mechanisms. It is believed that when a crack propagates through the matrix and reaches a GNP across its path, the crack gets deflected and absorbs some energy by creating a more tortuous path to release stress, resulting in toughening of the matrix.

**Table 2:** Mechanical properties of pure CS and GNP/CS composites.

Sample	Elastic modulus (GPa)	Micro-hardness (GPa)	Fracture toughness (MPa m^1/2^)	Brittleness index (µm^−1/2^)
CS	109.58±8.78	5.75±0.06	0.76±0.18	7.37
0.5 wt% GNP/CS	111.51±8.27	6.38±0.03	1.08±0.07	5.9
1 wt% GNP/CS	121.35±7.7	7.45±0.11	1.77±0.05	4.21
1.5 wt% GNP/CS	103.88±7.9	5.58±0.19	1.35±0.07	4.13
2 wt% GNP/CS	93.75±4.94	4.60±0.34	1.30±0.13	3.54

Brittleness index can be used to quantitatively assess the machinability of ceramics. Boccaccini reported that the good machinability occurs when the brittleness index of the ceramic is lower than 4.3 µ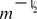 [31]. Meanwhile, the lower the brittleness index, the higher the machinability of the ceramics. As regarding the hardness and fracture toughness values in the present study, our results show that the GNPs are very effective in the BI of CS. In the case of 1 wt.% GNP/CS composite the BI value decreased from 7.37 to 4.21 µ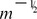, corresponding to a ∼40% decrease compared to pure CS. Another interesting observation is that the plot shows a systematic decreased in BI with increasing GNP concentration. Porwal *et al*. [46] reported that the graphene oxide nanoplatelets (GONP) has significant influence to reduce the BI and consequently improve the machinability performance in silica/GONP composites in comparison with pure silica. Overall, our results are in good agreement with results of Porwal *et al*. [46]

### *In vitro* HA forming ability

**Figure 7:**
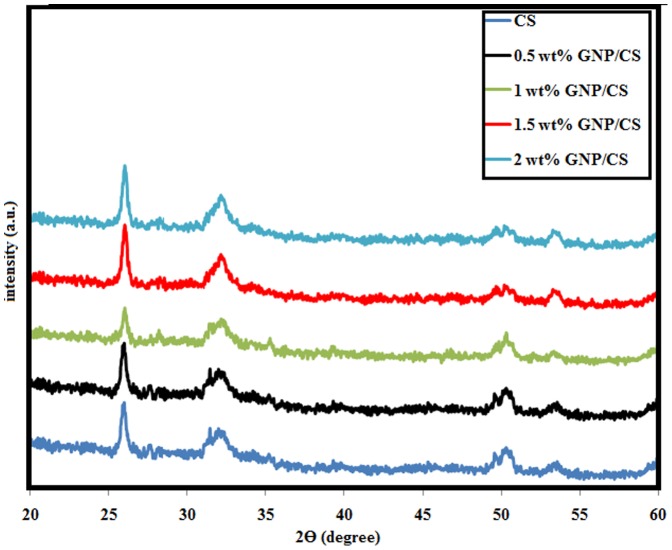
XRD patterns of pure CS and GNP/CS composites after soaking in the SBF solution for 14 days.

**Figure 8:**
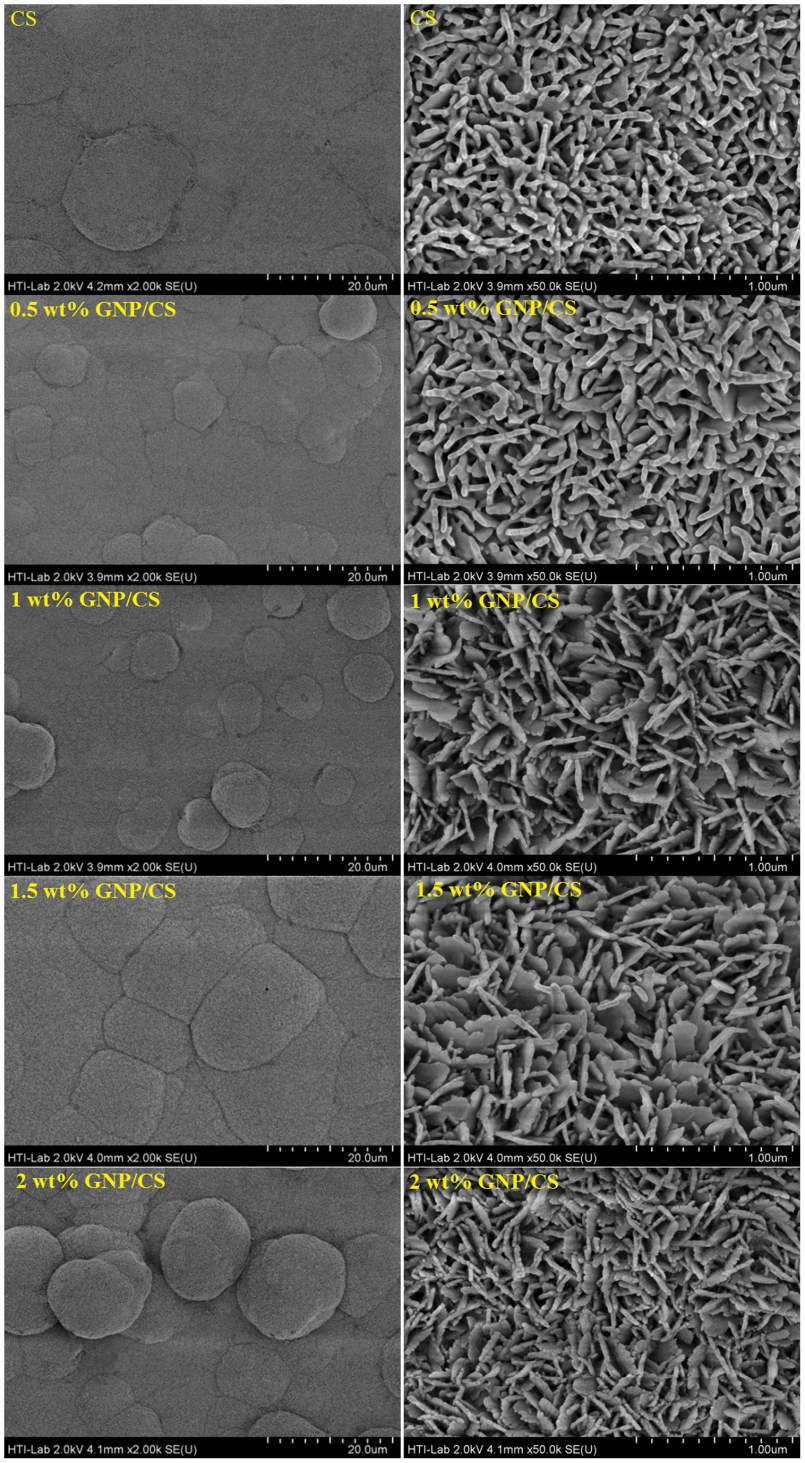
Low and high magnification FESEM images of apatite formation on pure CS and GNP/CS composites after soaking in the SBF for 14 days.

**Figure 9:**
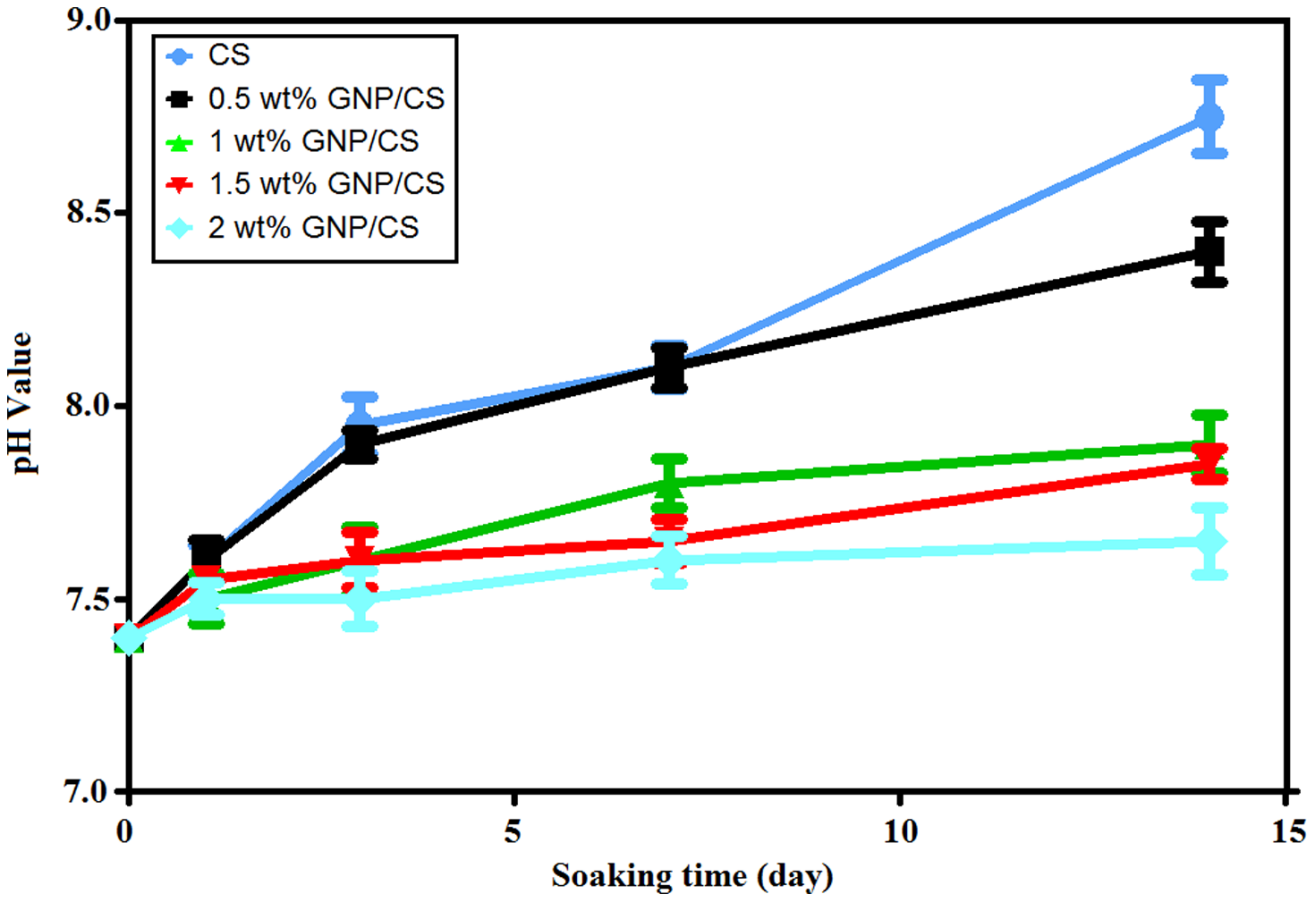
The change of pH value in SBF solution after soaking for various time periods.

The principal consideration for a biomaterial to be used for hard tissue replacement implants depends on two factors: good osseointegration of the implant in pristine bone, and admirable biocompatibility of the implant material for the growth promotion of osteoblast cells [47]. A homogeneous distribution and fast apatite formation rate implies a strong bone-bonding ability between the implant and surrounding tissues. Fig. 7 shows the XRD patterns of pure CS and GNP/CS composites after soaking in the SBF solution for 14 days. Only the characteristic peaks of hydroxyapatite (Standard Card No: JCPD 24-0033) were obvious and there was no difference in the intensity of peaks with different GNP contents in CS matrix after a prolonged soaking time of 14 days. All diffraction peaks of CS disappeared and broad peaks were detected at 2θ = 31.7°, 2θ = 49.5°, 2θ = 53.2°, with and a strong peak at 2θ = 26°, corresponding to the (2 1 1), (2 1 3), (0 0 4) and (0 0 2) planes of hydroxyapatite (HA), respectively. This suggests that more HA is formed on the surface of composites; and based from the shape of the peaks, this HA should be nanocrystalline in nature. Furthermore, no cristobalite or other peaks were detected in any samples soaked in SBF. Fig. 8 demonstrates the representative surface morphologies of pure CS and the GNP/CS composites after being soaked in SBF for 14 days. At low magnification, the apatite deposits on all samples showed typical spherical granules in density packed HA layers and the surfaces were fully covered by apatite. The higher magnification FESEM micrographs revealed that the morphology of mineralization product varies with addition of GNP into the CS matrix. Worm-shaped-like HA were formed on pure CS sample, whereas nano-sheet-like apatite forms on GNP/CS composites. Liu *et al.* [48] revealed that the mechanism of apatite formation on the CS surface involves dissolution of Ca^2+^ ions from the CS surface and leaving a Si-OH layer, which provided favourable sites for HA nucleation. On the other hand, the degree of supersaturation of the solution with respect to apatite increased with the dissolution of ions. Thus, the apatite nuclei were formed on the sample surface, and they spontaneously grew by consuming Ca^2+^ and 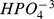 ions from the surrounding fluid. Fig. 9 shows that the incorporation of GNP into CS decreases the pH value in SBF solution compared to pure CS. Several research groups reported that the pH value has multiple effect on HBDC metabolism and function, where a pH value of 7.6 increased osteoblastic collagen synthesis, and is also critical factor for osteoporotic bone regeneration [49], [50]. The most significant and interesting finding is that when bone forms, the crosslinking of the collagen chains and the subsequent precipitation of HA are pH dependent and require an optimally alkaline pH at the bone formation site [49].

Liu *et al.* [48] believed that the pH value of the resultant SBF solution increased, due to the ionic exchange between calcium ions in CS and H^+^ in SBF. The results showed that, the value of pH of the SBF is higher over pure CS than over GNP/CS composites. For instance, the addition of 0.5 or 2 wt.% GNP into CS reduces the pH value of SBF from 8.75 to 8.40 and 7.65, respectively. This is because the graphene nanoplatelets have naturally occurring functional groups like ethers, carboxyls, or hydroxyls that can form acids and reduce the pH when the exposed GNPs are in contact with the SBF. On the other hand, the morphology of apatite formation depended drastically on the pH of the SBF solution [51]. Therefore, the difference in obtained morphologies between pure CS and GNP/CS composites is likely due to different pH values. These finding sufficiently indicates that the incorporation of GNP in CS decreased the pH value in SBF, suggesting a potential preferable material for *in vitro* bone cell culture. Our results also showed that GNP/CS ceramics sintered by HIP possessed good bioactivity and could develop a bone-like HA layer on their surface when soaked in SBF.

### Osteocompatibility characterization of GNP/CS composites by *in vitro* osteoblast culture

**Figure 10:**
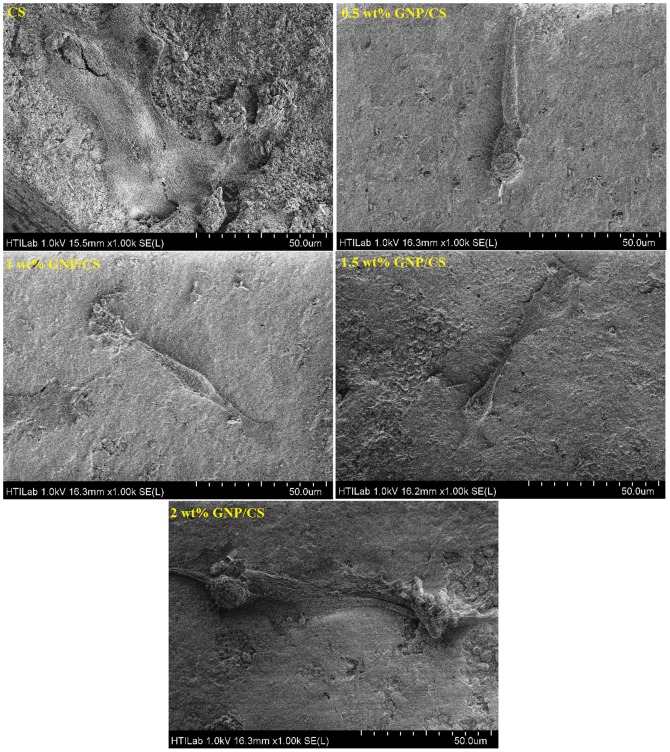
FESEM observations on cell morphology of hFOB osteoblasts cultured on pure CS and different GNP/CS composites after 24 hours. The scale bar in all the images is 50 µm.

The orthopedic implant is expected to promote cellular adhesion, proliferation, and differentiation. Once the osteoblasts cover the implant surface by proliferation and growth, they deposit collagen in the intercellular region, known as osteoids. Moreover, osteoblasts collect salt ions from the blood to release them on the osteoid matrix for mineralization and bone formation [52], [53]. This plays an essential role in osseointegration to determine the life-time of the implants [42]. Fig. 10 shows the cellular morphology of human fetal osteoblastic cell line (hFOB) cells grown on pure CS and GNP/CS composites matrices after 1 day of culture. The cells adhered and spread on the pure CS ceramic surface by means of thin cytoplasmic digitations as illustrated by the flattened morphology, and presented a close contact with the ceramic surface.

**Figure 11:**
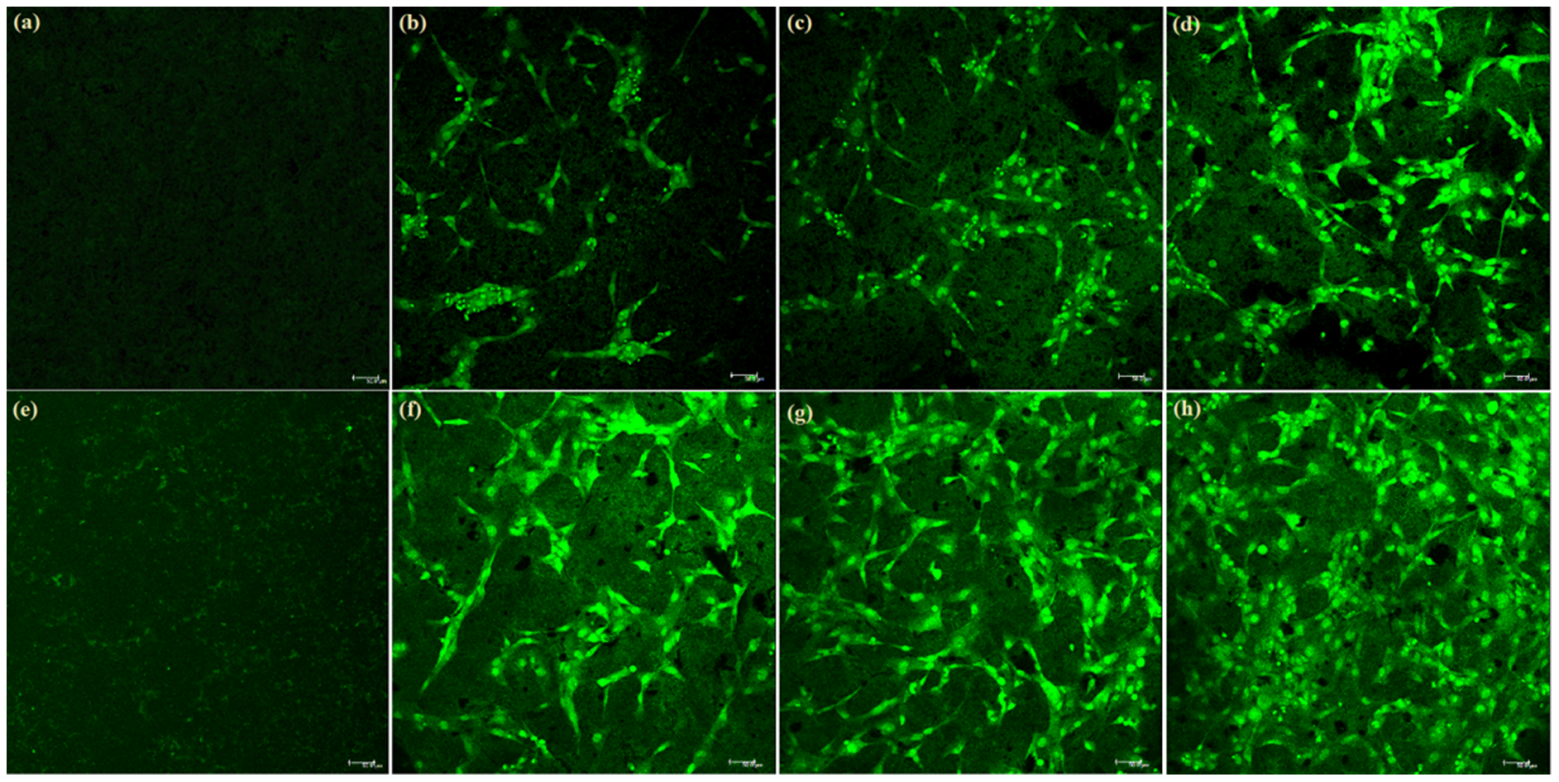
Comparison of the cell adhesion and proliferation on pure CS and 1 wt.%GNP/CS composite surfaces at different time points: (a) blank of pure CS, (b-d) 1, 3 and 5 days on pure CS discs, (e) blank of 1 wt.%GNP/CS, (f-h) 1, 3 and 5 days on 1 wt.%GNP/CS composite. The scale bar represents 50 µm.

Interestingly, it is noted that fibroblast-like shape and filopodia of the cells are observed on GNP/CS composites. Since an increased number of filopodia enabled the cells to tightly bind to GNP/CS surface; this composite is therefore considered more favorable to cellular integration than pure CS. Fig. 11 presents confocal laser scanning microscopy (CLSM) images of hFOB cells cultured on the surface of pure CS and 1.0 wt.% GNP/CS pellets. The osteoblast population clearly increases from 1 to 5 days on both surfaces. This observation indicates that CS and GNP/CS surfaces are suitable for osteoblast cell proliferation. Interestingly, the osteoblast population was visibly larger on GNP/CS surface than on pure CS after 3 and 5 days of culture as shown in Fig. 11.

**Figure 12:**
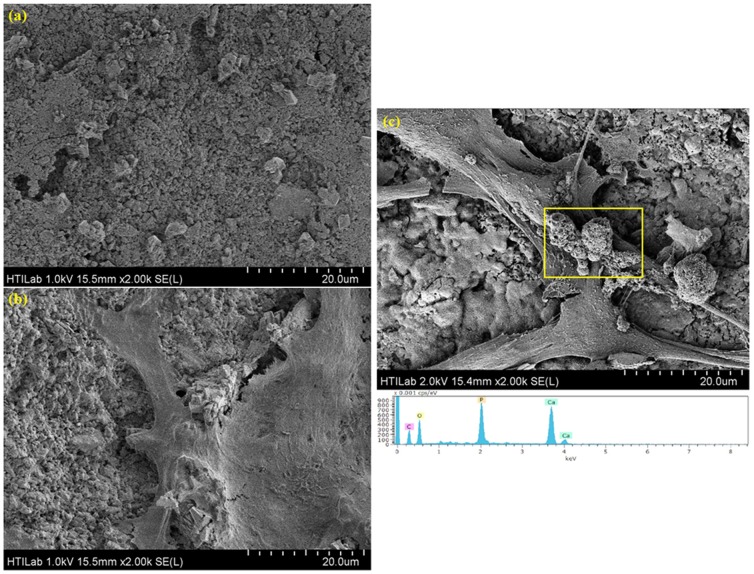
FESEM micrographs and the EDS spectrum of the hFOB cells. (a) 1 wt.%GNP/CS composite without cells, (b) Pure CS with hFOB cells,(c) FESEM micrographs and the EDS spectrum of the hFOB cells, indicating a significant presence of P and CS on the 1 wt.%GNP/CS composite following 3 days of seeding.

Merging of the cells promoted the formation of a rich ECM, showing high cell activity in the GNP/CS composites. The apatite formation on the ECM is important for mineralization and the generation of bone, as bone is formed by the mineralization of an organic matrix (largely collagen), through the nucleation and growth of a mineral closely similar to HA [54]. As shown in Fig. 12c, the mineral deposits present vivid apatite-like morphology and comprise fused globular aggregates of the minerals; and those granular minerals were illustrated in varying sizes on the 1 wt.% GNP/CS composite. While, as indicated in Fig. 12a and b, apatite like granules were not obsereved on cells surface of pure CS and blank control surface of pure CS without hFOB cells after 3 day of cell culture. It is known that mineralization refers to cell-mediated deposition of extracellular calcium and phosphorus salts where anionic matrix molecules take up the Ca^2+^, phosphate ions and serve as nucleation and growth sites leading to calcification [55]. Hence, the incorporation of GNP into CS is expected to have higher negative charge than pure CS in culture medium and leads to more rapid mineral deposition on the surface of osteoblasts. In addition, the EDS pattern of hFOB cells on the 1 wt.% GNP/CS composite indicated some presence of calcium and phosphate after 3 days seeding.

**Figure 13:**
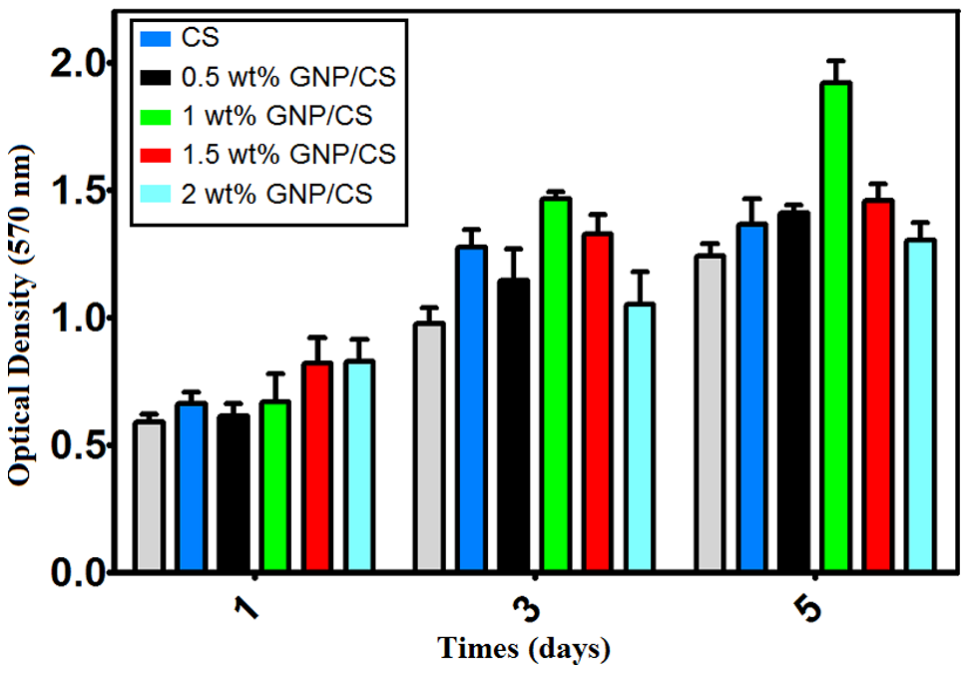
Proliferation of hFOB osteoblasts on different GNP/CS composites in comparison with pure CS and blank assessed using MTT assay (*p*<0.05,*n* = 5).

It is also of interest to note that the Ca/P molar ratio of the mineral deposit on the cells was 1.65, which is approximately equal to that of HA (1.67), suggesting that the apatite formed in the ECM primarily consisted of HA, which is also the major inorganic component of bone. On the other hand, since osteoblast cells are entirely responsible for creating bone tissue by producing osteoid (composed mainly of Type I collagen) before commencing the mineralization of the osteoid matrix, this observation would propose a clear relevance to the mechanism of collagen-based apatite mineral formation [56]. These results provide the first evidence of growth of the osteoblasts on GNP/CS composites and corroborated those quantitative results obtained shown in Fig. 13.

The cytotoxicity effects and cell proliferation of osteoblast cells on the various samples are shown in Fig. 13 for comparison. There was no cytotoxicity of GNP/CS composites found in the hFOB cell line through the MTT assay. The cell proliferation of hFOB cells on each sample increased with the extension of culture time. The highest amount of cells could be observed on the 1 wt.% GNP/CS composite. Many reports [2], [7], [48],[57] have already shown that ionic dissolution products from CS are key factors in the metabolism, proliferation, cell–cell and matrix-cell adhesion of osteoblasts. Meanwhile, Shen *et al.* [50] found that the pH value of strontium-containing CS is a critical factor for the proliferation and alkaline phosphatase (ALP) activity of osteoblasts. Our results indicated a decrease in the pH of SBF due to increased GNP content in the CS ceramic, which can be desirable for cell growth. Furthermore, smaller grain size generally results in more specific area of the sample, which ultimately increases dissolution of calcium ions, and therefore it can be promoted better osteoblast interactions [23], [58], [59]. The MTT result shows that, the number of cells on 1 wt.% GNP/CS composite with finer grain size was significantly higher than those on Pure CS and 0.5, 1.5 and 2 Wt.% GNP. On the other hand, researchers have studied the effect of concentration of graphene on cell viability as well as cell cytotoxicity. They have concluded the cell viability can be affected by concentration of graphene [60], [61]. Previous studies have also indicated that graphene incorporation into hydroxyapatite stimulated osteoblast proliferation [62], [63], and the cytotoxicity of graphene to osteoblast is concentration-dependent and lowering the concentration of the graphene fillers results in improved biocompatibility to bone cells [64]–[66]. Our results showed that GNP incorporation into CS has a positive effect on the proliferation of hFOB cells, while the degree of proliferation was related to different GNP-containing CS ceramics. These results are a good indication that the GNP/CS composites are suitable to support the biocompatibility in terms of cell proliferation. However, it should be noted that the *in vitro* results we report here are very preliminary and further comprehensive understanding about the biocompatibility of the novel GNP/CS composites is required. Particularly, implantation in bone tissue and for longer period is required for absolute assessment of *in-vivo* biocompatibility, in order to establish the feasibility of employing GNP/CS in orthopedic implants and other tissue engineering applications.

## Conclusions

GNS-reinforced calcium silicate composites have been fabricated by HIP and the influence of the different amounts of GNP (0.5, 1, 1.5 and 2 wt.%) on the microstructure development and mechanical properties were investigated. The incorporation of GNP into CS has a significant effect on grain size. Grain size is reduced in 0.5 and 1 wt.% GNP as GNP might tend to wrap around grains and inhibit grain growth. Compared to pure CS, the 1 wt.% GNP/CS composite displayed an increased hardness and ∼130% and ∼40% improvement in fracture toughness and brittleness index. Crack bridging, pull-out GNP, crack branching and crack deflection have been observed and are believed to be the causes of increased toughness. The SBF soaking results revealed that the GNP/CS composites have apatite-forming ability. Our results indicate that the incorporation of 1 wt.% GNP into CS stimulated hFOB cells, as opposed to cell seeding onto pure CS ceramics. The results in this study demonstrate promising *in vitro* cell compatibility and bioactivity of GNP/CS biocomposites and their potential applications for load-bearing biomaterials.
